# Left sleeve pneumonectomy performed through a sternotomy incision with cardiopulmonary bypass for bronchogenic carcinoma: report of two cases

**DOI:** 10.1186/1749-8090-8-S1-P136

**Published:** 2013-09-11

**Authors:** Ts Minchev, E Manolov, V Marinchev, I Todorov, V Georgiev, I Stoimenov, G Kirova, P Dakova

**Affiliations:** 1 Thoracic Surgery, Tokuda Hospital, Sofia, Bulgaria; 2 ICU, Tokuda Hospital, Sofia, Bulgaria; 3 Medical Imaging, Tokuda Hospital, Sofia, Bulgaria; 4 Pathology, Tokuda Hospital, Sofia, Bulgaria

## Background, methods and results

We report herein the cases of two patients with bronchial carcinomas in the left main bronchus who were successfully treated by left sleeve pneumonectomy performed through a sternotomy incision for the first time in Bulgaria. Adequate oxygenation during tracheobronchial anastomoses was achieved by cardiopulmonary bypass in both patients. Tracheobronchial anastomosis was relatively easy to perform in the excellent operative field achieved by this method. The postoperative courses of both patients were uneventful. Was no need for prolonged mechanical ventilation.

## Conclusion

The procedure presented in this paper is considered to be a safe and effective method of performing left sleeve pneumonectomy.

